# A Psychoanalytic-Derived Brief Psychotherapeutic Approach in the Treatment of Major Depression: Monotherapy Studies

**DOI:** 10.3390/medicina58101335

**Published:** 2022-09-23

**Authors:** Gabriele Di Salvo, Matteo Bianco, Elena Teobaldi, Giuseppe Maina, Gianluca Rosso

**Affiliations:** 1 Department of Neurosciences “Rita Levi Montalcini”, University of Turin, 10126 Turin, Italy; 2 Psychiatric Unit, San Luigi Gonzaga University Hospital, 10043 Turin, Italy

**Keywords:** short term psychodynamic therapy, STPP, major depressive disorders, monotherapy, recurrency

## Abstract

Over the years, short term psychodynamic therapy (STPP) has been broadly researched in order to evaluate its efficacy in the treatment of major psychiatric disorders. In particular, a consistent number of studies focused on assessing clinical outcomes of the principal psychodynamic techniques in treating depressive disorders. We conducted a narrative review in which we aimed to evaluate the efficacy of STPP in monotherapy in major depressive disorder and to assess possible features that may correlate with its clinical use. Databases searched were PubMed, Ovid, Scopus, PsycINFO and Cochrane Libraries from inception to July 2022. Our research underlined that STPP in monotherapy is particularly effective in moderately severe depression and in preventing depressive relapses. Moreover, a case-by-case evaluation of its efficacy should be performed when considering STPP for the treatment of major depression with other comorbid psychiatric conditions. Although such key points emerged from scientific evidence, STPP should be better studied in the long-term perspective; further research is needed to define the clinical scenarios in which STPP can be considered a first-line approach as monotherapy in major depressive disorder compared to medications or other types of psychotherapy.

## 1. Introduction

### 1.1. Short-Term Psychodynamic Psychotherapy (STPP): Origins and Theoretical Foundation

The term Short-term psychodynamic psychotherapy (STPP) identifies a set of psychotherapeutic techniques-rooted in the Freudian theoretical model [1] that developed over time newer and specific methodological approaches, progressively departing from classical long-term models. STPPs largely refer to basic principles of psychoanalysis: clinical symptoms are considered as expression of conflicts between unconscious psychic instances and the occurrence of pathologies is linked with relational dysfunction in early development and object relations. Furthermore, therapeutic work is carried out through the analysis of verbal and non-verbal communication and transferential/countertransferential dynamics [2].

At the same time, STPPs embody the need for change of traditional models in response to historical and social progresses, which demanded for adjustments in psychotherapeutic interventions: expanding the fields of application, identifying areas of intervention by determining indications and contraindications, verifying results and evaluating costs/benefits [2,3].

The first reason for differentiation from classical models was certainly represented by the request for a limitation in the number of sessions and the overall duration, which at present constitutes one of the founding features of STPPs. The need for brevity of treatment, in opposition to the tendency to increase the duration of analysis, was firstly postulated by Ferenczi and Rank [4], who questioned the centrality of the elaboration of infantile neurosis and the consequent development of personality as fundamental paths for therapeutic change. The authors argued that the analyst’s attention should be focused on the emotional elements reproduced by the patient through the relationship with the therapist (i.e., present transfert), since relational dynamics of the past tend to repeat in the present. Alexander and French [5] proceeded in challenging the belief that short-term therapies could not lead to lasting transformation, emphasizing the concept of recovery occurring outside the therapy session rather than during the analysis.

The second reason for evolving beyond classical models can be identified in the request for more versatility towards different types of patients: frequently, subjects with severe psychic disorders could not tolerate a classical psychoanalytic treatment. The overcoming of the theoretical and clinical Freudian psychoanalytic orientation was especially carried out through the interpretative-applicative models proposed by the American literature. In particular, Luborsky performed comparative studies on psychotherapies which underlined the role of transference as an essential working tool for the management of emotional conflicts. By identifying a core conflictual relationship theme (CCRT) as foundation of supportive-expressive therapies, Luborsky suggested current relational issues of the individual as the target of short-term psychodynamic interventions, focusing the gain of insight towards recurring conflicts (both intrapsychic and interpersonal) rather than to more classical psychoanalytic elements [6]. This model led to a problem-centered psychotherapeutic approach, therefore particularly suitable to a time-limited therapeutic setting such as the public health care system. In this regard, the introduction of a manual of psychoanalytic psychotherapy proved to be a valuable asset, both for the training of practitioners and for research development: therapists were provided a basic framework to measure elements associated to the internal coherence and the outcome of psychotherapeutic practice [7]. In fact, the same author emphasized the need to obtain clearer parameters to determine a patient’s therapeutic improvement. Three areas were identified:

- changes in the patient (better understanding of symptoms and conflicts, internalization of the alliance and ability to consciously manage the problem);
- modalities of change in the patient (active engagement, elaboration of relevant problems, ability to establish a therapeutic alliance);
- means used by the therapist (facilitation of expression, comprehension and reworking, ability to provide useful elements to integrate the patient’s information).

Beyond the historical path of the various schools of thought (Table 1), it is necessary to highlight some common aspects shared by the different STPP techniques. The concept of time has now become an integral part of the interpersonal relationship, since the brevity of treatment holds major importance in accelerating the psychotherapeutic process. In this regard, anxiety and pain for separation-in contrast with the desire for continuity and repetition-can increase the emotional tension experienced in the context of therapy itself [8]. Such distress can be exploited in a brief psychodynamic intervention to provide the tools to help patients showing anxiety, depression and interpersonal difficulties, which are considered the product of chronic maladjustments [9]. In this field, the main techniques are represented by interpretation and clarification: the therapist has thus the task of highlighting connections with significant interactions from the past, comparing them to the present relationship.

These models are therefore based on techniques that favor a significant reduction in the time of intervention, foregoing the complete restructuring of personality in favor of limited but not negligible objectives, such as clinical improvement and social-relational functioning, contact with emotional experiences and cognitive acquisition of conflicts and limitations [10].

Another crucial feature of STPP is the setting negotiation: it is necessary to define both the area in which the therapy will be developed and the frequency and duration of meetings (more frequently weekly, 45 min each). The total number of sessions generally varies from 10 to 30 (12 according to Mann’s school), ranging from 7 to 40; in some circumstances ultra-short techniques (less than 6 meetings) may be applied. Particular attention is paid to the definition of the focus, which must address a precise therapeutic theme towards which the intervention is targeted. The content of the focus can be equally represented by a symptom, conflicts, maladaptive or critical preconscious situations or to grief and separation-related issues [8].

Lastly, the therapist has to seek an effective management of the therapeutic alliance, with moderately active and empathic involvement or, according to the model of the American schools, with an aggressive and deliberately provocative attitude to induce an intense motivational reaction in the patient [11,12].

In addition to the technical and ideological motivations that brought to the development of different techniques, the debate around psychotherapy has been centered on public assistance and hospitals as pivots of psychiatric care [13,14,15]. Interest towards more detailed treatment modalities was motivated by various factors: growing need for applicability of treatments in the public health care system, demands for cost containment, and lack of scientific studies documenting efficacy, safety, appropriateness, and cost-effectiveness of therapies [9].

In response to the need for evaluation of effectiveness, validity and reproducible and scientifically comparable models, in the last decades psychotherapy has been object of systematic research. A number of studies have evaluated the clinical efficacy in treating major psychiatric disorders [16,17,18,19,20] and verified the superiority of these therapies in sample groups compared to controls or placebo [21]. Other studies have evaluated the psychodynamic approach by examining possible predictive factors of outcome and selecting the most suitable techniques according to the characteristics of the patients [22,23]. Despite such caution, the relationship between this type of therapeutic intervention and its effects on psychiatric disorders still requires further evaluation [24].

The choice of psychotherapeutic intervention undergoes rigorous patient selection criteria. The indications for treatment are not only limited to the clinical characteristics of the disorder: other variables such as patient’s insight, level of education, motivation for change, relational skills and available economic resources are also taken into account.

Currently, STPP find application in Depressive disorders, Anxiety Disorders, Eating Disorders (especially in Bulimia Nervosa), stress-related disorders and occasionally in Somatoform Disorders and substance use disorders [25,26]. The efficacy of the treatment in Personality Disorders has not yet provided definitive data. Absolute contraindications to treatment are current psychotic symptoms, drug addiction and personal traits that may interfere with the therapeutic relationship.

### 1.2. STPP in the Treatment of Major Depressive Disorder (MDD)

In recent years, the therapeutic approach to MDD has increasingly embraced the use of psychotherapeutic treatment, both in association with pharmacotherapy and in monotherapy. The growing need for more flexible models arised alongside the first results of controlled studies that showed high rates of non-response and relapse in patients treated with antidepressant monotherapy [27], as confirmed by more recent research [28]. Moreover, such necessity for versatility is also strengthened by the modern conception of depression as a spectrum of disorders, whose clinical presentation depends on multiple emotional, psychomotor, cognitive, personality and somatic aspects [29]. In particular, depression can lead to somatic changes as well as psychic symptoms: it is widely acknowledged that somatic symptoms predict worse prognosis in MDD independently of psychiatric characteristics, medical comorbidities, lifestyle and disability, especially if cardiopulmonary and gastrointestinal systems are involved [30]. Somatic symptoms are also related to biological structural changes of the brain, feature which furtherly contributes to the complexity of depression [31].

Given these premises, the brief psychodynamic approach targets a substantial modification of the substrate of the depressive disorder, focusing on the therapeutical relationship and on a series of consecutive, timeframe-related goals:

1. decreasing intensity of symptoms through expression of suppressed negative feelings;
2. modulation of Super-Egoic standards of perfection, in order to reduce feelings of inappropriateness and guilt and to strengthen self-esteem;
3. increasing awareness on the patient’s current interpersonal relationships [32].

Concerning MDD, guidelines recommend psychotherapy in monotherapy as a first line in case of mild to moderate depressive symptoms, while a combination of medication plus psychotherapy is suggested in case of moderate to severe symptomatology [33,34]. Therefore, most of the studies on STPP in patients with MDD are focused on combined approach [35,36,37,38,39] and only few evaluated STPP monotherapy [40,41]. Regarding ‘minor’ depressive disorders, there are few evidence concerning STPP monotherapy as well [42].

Furthermore, according to the APA guidelines [43] psychodynamic psychotherapy is particularly indicated in patients with MDD with specific characteristics such as the presence of chronic feeling of emptiness, rigid self-expectations and lack of self-worth, history of childhood abuse, loss or separation or chronic conflict in interpersonal relationship. Moreover STPP, as other psychotherapeutic strategies, has to be preferred as a first line in case of MDD during pregnancy, post-partum, childhood and adolescence [33].

Given the above, STPP in monotherapy has less evidence than cognitive-behavioural therapy (CBT) or interpersonal therapy (ITP), which are thus the first two psychotherapy approaches recommended by the most recent guidelines [34,44,45,46].

However, the time-limited and problem-focused approach of STPP can make it more suitable in the public health context compared to other psychotherapeutic techniques.

The purpose of the present paper is to critically review the current knowledge on the efficacy of STPP in monotherapy in the treatment of MDD.

## 2. Methods

We conducted a narrative review of published articles on the treatment of MDD with STPP.

Searches were made in a range of scientific databases (PubMed, Ovid, Scopus, PsycINFO, Cochrane Library) from inception to June 2022. The search terms “short-term dynamic psychotherapy”, “brief dynamic psychotherapy”, “STPP”, and “BDT” were combined, using the boolean AND, with “depressive spectrum disorders”, “unipolar depression”, “major depressive disorder”, “MDD” and “monotherapy”. Then, a manual search for references lists from articles selected in the previous search was done. The inclusion criteria for this narrative review were as follows: (a) participants diagnosed with unipolar depressive spectrum disorders; (b) participants treated with STPP in monotherapy; (c) outcome clearly defined in terms of STPP effectiveness. Articles were assessed for inclusion at three stages: title screening, abstract screening, and full text screening. Three reviewers (GDS, MB and ET) independently decided which articles to include according to clinical importance and eligibility criteria. In case of disagreement, the senior authors (GR and GM) were consulted to mediate consensual decision.

## 3. Results

**Figure 1:**
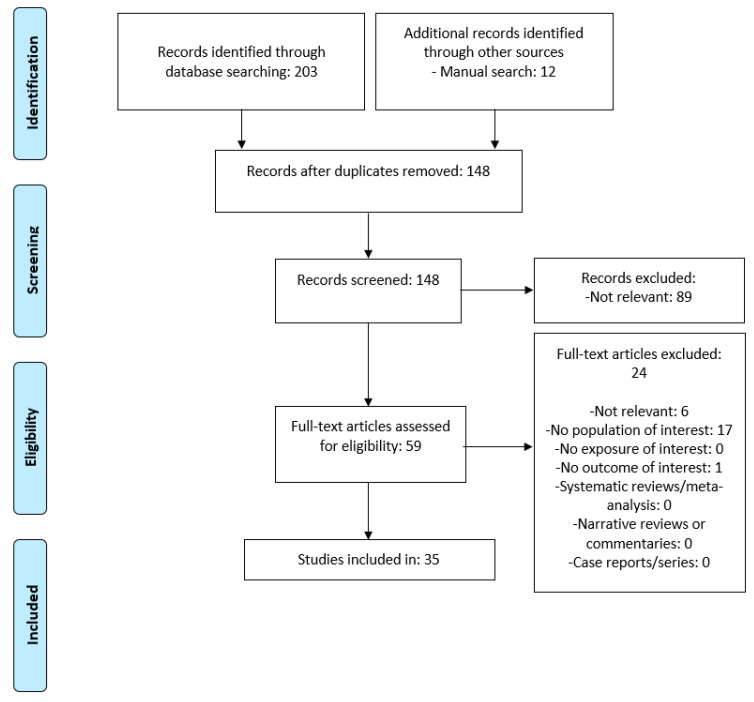
Flow diagram of the narrative review.

A flowchart of studies selected and included in the narrative review is provided in Figure 1.

As mentioned before, initial research on STPP aimed mainly to identify eligibility characteristics of patients and to define the therapeutic relationship [46,47,48,49,50].

Later, some open-label uncontrolled studies have showed the efficacy of STPP in improving depressive symptoms in patients with MDD [17,19,51,52,53,54]. Hilsenroth and colleagues replicated and extended these earlier findings with a more rigorous study: 21 outpatients with MDD underwent a 30-meeting cycle of STPP and a significant improvement in depressive symptoms and interpersonal, social and occupational functioning, measured on both semistructured clinical interviews and self-administered questionnaires, was detected in 80% of those subjects who completed the study [55].

More recently, a few controlled trials have been performed, in order to compare the efficacy of STPP with other treatment options (pharmacotherapy or other variant of psychotherapy) in MDD.

Salminen and colleagues conducted a comparative study on the efficacy of STPP versus fluoxetine in patients with mild or moderate episode of MDD. Fifty-one subjects were randomized to receive either STPP (1 session/week) or fluoxetine (20–40 mg/day) for 16 weeks: among the patients who completed the follow-up, 57% in the STPP group and 68% in the fluoxetine group achieved full remission after 4 months, showing a comparable efficacy of these two treatment forms [40]. This finding was confirmed by two further trials [56,57], which did not find significant difference between STPP and antidepressants in the treatment of acute depressive episodes.

Concerning the comparison between STPP and other psychotherapeutic techniques, data analysis initially reported significantly lower effectiveness of brief psychodynamic techniques compared with cognitive and behavioral therapies [16,18], while more recent literature suggests comparable outcomes. In particular, Leichsenring’s meta-analysis on a total sample of 416 individuals evaluated the effectiveness of brief psychodynamic therapy in comparison with cognitive and behavioral therapies; six clinical trials performed on outpatients diagnosed with MDD were evaluated. Data showed different rates of clinical improvement and remission, largely dependent on the type of criteria applied in each study and partly on the timing of evaluation: STPP determined an effective post-treatment response in 45–70% of the patients, while stable improvement was observed in 26–83% of the sample at subsequent follow-ups. No significant differences emerged between the different psychotherapy models used, regarding either the sub-sample with post-treatment clinical improvement or subsequent follow-up assessments [58].

More recently, a randomized clinical trial authors compared the efficacy of STPP with that of CBT, analyzing 341 patients with a major depressive episode randomly assigned to 16 sessions of individual STPP or CBT [59]. The primary outcome measure was post-treatment remission rate (HAM-D score < 7), while the secondary outcome included mean post-treatment HAM-D score, patient-rated depression score and 1-year follow-up outcomes: no statistically significant treatment differences were found between the two subsamples for any of the outcome measures, but noninferiority of STPP could not be demonstrated for post treatment remission rates or any of the follow-up measures.

Though STPP is a supported treatment for depression frequently applied in clinical practice, studies often have small sample sizes and there is a paucity of high-quality, rigorous controlled trials. Then, it remains open to debate which patients, which subtypes of depression and which illness phases can benefit most from this therapy [35].

Analyzing available literature, we have identified some key points that can lead to a more specific and personalized use of STPP monotherapy in MDD, as well as to a more accurate assessment of outcomes.

(1) STPP efficacy in MDD, compared with other treatment strategies, is more apparent at long-term follow-up rather than in the immediate post-treatment period.

In a large meta-analysis, Driessen and colleagues found that at the end of the acute phase other psychotherapeutic treatments were superior to STPP, while no significant differences were found at 3-month and 12-month follow-up: improvements given by STPP were found to be maintained or even increased at follow-up assessments [60]. Remarkable long-term results were found by Maina and colleagues in a RCT performed on 30 patients diagnosed with minor depressive disorder, which compared STPP and generic supportive therapy with a control group (patients in the waiting list): while both STPP and supportive therapy led to improvement of symptoms in the short term, STPP was more effective at follow up evaluation at 6 months [42]. Such findings were confirmed by Shedler, who analyzed five meta-analyses showing that psychodynamic psychotherapy leads to lasting and improving-over-time benefits, even after treatment end. Moreover, in his work Shedler highlighted the importance of psychodynamic processes in predicting successful outcomes even in non-psychodynamic therapies, since skilled practitioners often utilize techniques that are based on psychodynamic core principles, regardless of the psychotherapeutic approach [61]. On the other hand, having low-quality interpersonal relationships before starting STPP was found to be a negative predictive factor to achieve stable long-term dynamic changes, while patients with high-quality interpersonal relations had more favorable dynamic outcomes with a brief treatment approach (10–25 sessions) [62].

(2) STPP may be more effective in moderate than in mild depression.

Although the potential correlation between STPP efficacy and depression severity might be a major point of interest to direct treatment strategies, literature about such topic is scarce. A study performed by Rosso and colleagues addressed the question of the efficacy of STPP in depressive disorders in correlation with symptoms severity [41]. In this RCT, STPP was put in comparison with brief supportive psychotherapy (BSP), analyzing 88 outpatients with depressive disorders. In the subsample of subjects with mild depressive disorders, no statistically significant differences emerged between the two treatments on all efficacy measures; conversely in the subgroup of patients with moderate depressive disorders, the remission rates of patients treated with STPP were higher than those of patients treated with BSP at 6 months of follow-up. In conclusion, the study suggests that the benefit of STPP in MDD is higher in moderate than in mild depression.

(3) In case of depression with concomitant psychiatric comorbidities, the efficacy of STPP must be evaluated on a case-by-case basis.

A few studies in the literature examined the effectiveness of STPP in treating MDD with other concurrent psychiatric conditions; still, no consensus has been reached on its use in such cases. For example, a study conducted on ODC patients by Maina and colleagues showed no significant impact of STPP in treating either depressive or obsessive symptoms [38]. Even though the study was performed on patients already on antidepressants, it is noteworthy how a comorbid condition can considerably influence the treatment outcome, given the efficacy of STPP in treating MDD per se. Other conditions, such as personality disorders, have been researched alongside MDD in the clinical evaluation of STPP. Leichsenring and colleagues, in their empiric methodological review of psychodynamic psychotherapies in depressive disorders, found a twofold risk for poor outcome when MDD was co-diagnosed with a personality disorder, although some limitations emerged in the included studies (primarily targeting personality aspects during the course of treatment, different sample sizes, including different personality clusters) [63]. A meta-analytic work by Abbass and colleagues sought to deepen the efficacy of STPP in comorbid MDD and personality disorders: contrarily, no differences were found when STPP and other psychotherapeutic approaches were compared and symptom improvement was maintained over a mean 1.5 year follow up period [64]. Lastly, Driessen and colleagues compared the efficacy of STPP and Cognitive-Behavioural Therapy (CBT) in patients with depressive disorders; by design, subjects with severe depressive symptoms were given antidepressants + STPP or CBT, while other patients received psychotherapy only. STPP was found more efficacious among moderately depressed patients (undergoing therefore STPP only) who showed low baseline comorbid anxiety levels: such patients may have benefited from STPP as they are speculated to better experience an open relational- and insight-oriented dynamic dialogue, while they feel less comfortable with a structured and protocolized approach like CBT [65].

(4) STPP is effective in preventing MDD recurrences.

In mild forms of depression, psychotherapy has been shown to be equally effective to pharmacotherapy at the end of the acute phase of treatment in terms of symptom remission, but superior in the long term, especially with regard to relapse prevention [66,67,68]. A large quantity of the papers currently published about STPP focused on evaluating its effectiveness in preventing depressive recurrences, as well as validating its effectiveness during acute phases. A Cochrane Database meta-analysis examined the effects of STPP across several mental disorders, including MDD, showing how a significant improvement is maintained on medium- and long-term follow up [69]. Such data is confirmed by other well-designed meta-analytic works, such as Driessen and colleagues’, which observed consistent long-term efficacy of STPP in MDD, especially at 3-month, 6-month and 12-month evaluations [70]. One study in particular proposed to validate STPP efficacy through a longer follow up period. Rosso and colleagues retrospectively evaluated the recurrence rates during a 5-year treatment-free period in a sample of patients with first-episode depression, treated in the acute phase either with STPP or antidepressants: 71.7% of remitters to STPP did not experience depressive recurrencies, compared to 46.8% resulting from patients priorly treated with pharmacotherapy. An additional significant result was the rate of onset of hypomanic/manic episodes during the observation period, significantly higher in remitters to antidepressants than those treated with STPP (9.2% vs. 2.2%) [71]. Such results match the data from Koppers and colleagues, who detected at the same cutoff (five years) a recurrency rate of 37% in patients who underwent STPP in monotherapy, with no significative beneficial effect in the long run given by a combined approach with antidepressants (44% recurrency rate); moreover, being a young female was detected as a possible predictive factor for recurrencies [72].

## 4. Discussion

The aim of this paper was to review literature data on the efficacy of STPP monotherapy in the treatment of MDD.

Although STPP monotherapy is widely considered an effective therapeutic option for patients with MDD, the empirical support for this statement is still small. Indeed, the majority of available data derive from open-label studies, while there is a lack of controlled trials conducted on large samples with rigorous methods: the evidence on STPP efficacy compared with other psychotherapeutic techniques and with pharmacotherapy is still weak, both concerning the acute phase and the recurrence prevention.

This may explain why, in international guidelines, STPP ranks second to other psychotherapeutic approaches such as CBT and ITP, which have stronger evidence. Analyzing available data, despite the above-mentioned limitations, STPP appears to be a valid treatment strategy for MDD, especially in preventing affective recurrences and in improving the long-term outcome. The STPP property to enhance the patient’s insight into repetitive conflicts and trauma and to provide a corrective emotional experience might be a specific therapeutic factor sustaining the patient’s improvements not only during treatment sessions, but also in the long-term period [36,37,42].

On the other hand, further understanding of how depression responds to STPP is awaited to direct treatment strategies, moving toward a more tailored treatment. The correlation between the STPP effect and the severity of depressive symptoms is still little explored in literature: the findings that the benefit of STPP in treating depressive symptoms is stronger in moderate than in mild depression [41] need to be confirmed by other studies conducted in larger sample sizes and with longer follow-up periods. Furthermore, given the paucity of data on the effectiveness of STPP in treating MDD with other concurrent psychiatric conditions, no consensus has been reached on its use in such cases. Lastly, to the best of our knowledge, no study has been specifically designed to address the question of which depression subtypes are more likely to benefit from STPP.

## 5. Conclusions

In conclusion, STPP in monotherapy can potentially make significant contributions in the treatment of MDD, also given its applicability in public health services. Still, the current literature lacks rigorous studies and the impact of crucial clinical factors on the efficacy of STPP, such as the age of onset of depression, the duration of illness and the duration of untreated illness, has not been investigated. For such reasons, short and long-term controlled studies on large samples comparing STPP with other psychotherapies and pharmacotherapy are awaited; moreover, further research is needed to evaluate whether specific subgroups of subjects might find STPP more beneficial and whether specific clinical features can be used as a guide in selecting treatment for every patient.

## Figures and Tables

**Table 1:** Comparison of Short term psychodynamic therapy schools.

	Malan	Sifneos	Davanloo	Mann
Selection criteria	Yes	Strict	Yes	Broad
Number of meetings	20–40	12–20	5–40	12
Predetermined endpoint	Yes	No	No	Yes
Active therapist	Yes	Very active	Very active	Yes
Neutrality	Yes	No	No	No
Focus	Yes	Yes	Yes	Yes
Transference interpretation	Yes	No	Yes	Yes
Targeting defence mechanisms	No	Yes	Yes	No
Confrontational approach	No	No	Yes	No
Suggestive approach	No	No	No	Yes
Pedagogical approach	No	Yes	No	Yes
Relaxation techniques	No	No	No	No
Pharmacological treatment	No	No	No	No

## Data Availability

Not applicable.
